# Module-Designed Carbon-Coated Separators for High-Loading, High-Sulfur-Utilization Cathodes in Lithium–Sulfur Batteries

**DOI:** 10.3390/molecules27010228

**Published:** 2021-12-30

**Authors:** Yi-Chen Huang, Yin-Ju Yen, Yu-Hsun Tseng, Sheng-Heng Chung

**Affiliations:** 1 Department of Materials Science and Engineering, National Cheng Kung University, No. 1, University Road, Tainan City 701, Taiwan; n56094449@gs.ncku.edu.tw (Y.-C.H.); n56094261@gs.ncku.edu.tw (Y.-J.Y.); n56094295@gs.ncku.edu.tw (Y.-H.T.); 2 Hierarchical Green-Energy Materials Research Center, National Cheng Kung University, No. 1, University Road, Tainan City 701, Taiwan

**Keywords:** lithium–sulfur batteries, electrochemistry, porosity, carbon, polysulfides

## Abstract

Lithium–sulfur batteries have great potential as next-generation energy-storage devices because of their high theoretical charge-storage capacity and the low cost of the sulfur cathode. To accelerate the development of lithium–sulfur technology, it is necessary to address the intrinsic material and extrinsic technological challenges brought about by the insulating active solid-state materials and the soluble active liquid-state materials. Herein, we report a systematic investigation of module-designed carbon-coated separators, where the carbon coating layer on the polypropylene membrane decreases the irreversible loss of dissolved polysulfides and increases the reaction kinetics of the high-loading sulfur cathode. Eight different conductive carbon coatings were considered to investigate how the materials’ characteristics contribute to the lithium–sulfur cell’s cathode performance. The cell with a nonporous-carbon-coated separator delivered an optimized peak capacity of 1112 mA∙h g^−1^ at a cycling rate of C/10 and retained a high reversible capacity of 710 mA∙h g^−1^ after 200 cycles under lean-electrolyte conditions. Moreover, we demonstrate the practical high specific capacity of the cathode and its commercial potential, achieving high sulfur loading and content of 4.0 mg cm^−2^ and 70 wt%, respectively, and attaining high areal and gravimetric capacities of 4.45 mA∙h cm^−2^ and 778 mA∙h g^−1^, respectively.

## 1. Introduction

Conventional lithium-ion batteries apply composite insertion electrodes to generate high and stable charge-storage capacities; this approach dominates the commercial market of energy-storage devices. Materials and fabrication processes have improved steadily over the past thirty years, but have now reached the theoretical limitation of lithium-ion technology [1,2,3]. Thus, the rapidly growing demand for sustainable renewable energy and the emerging markets of electric vehicles and energy-storage plants have motivated the exploration of advanced energy-storage technologies. For instance, these developments could enable rechargeable batteries to adopt inexpensive active materials to attain high energy densities far beyond those of current lithium-ion batteries [1,3,4,5,6,7]. Among the promising energy-storage candidates, the electrochemical lithium–sulfur battery has attracted great attention as the most desirable next-generation energy-storage system because it combines high energy density and affordable costs with minimal environmental impact [4,5,6,7]. Lithium–sulfur batteries are expected to provide gravimetric energy density of 400–600 W∙h kg^−1^ (approximately double that of lithium-ion batteries), along with volumetric energy density of 700 W∙h L^−1^ for a fully packaged system [4,5,6,7]. This is because the high-capacity sulfur cathode used in lithium–sulfur batteries is not only abundant and environmentally benign, but also offers an order of magnitude higher charge-storage capacity compared with the composite insertion electrodes used in current lithium-ion technology [4,5,6,7].

To commercialize lithium–sulfur batteries, increasing efforts have been devoted to addressing the intrinsic material and extrinsic technological challenges, which are caused by the low electronic conductivity of active solid-state materials [7,8,9] and the poor electrochemical stability of active liquid-state materials [10,11,12]. The intrinsically low electronic conductivity of the active solid-state materials (i.e., sulfur and its end-discharge product, lithium sulfide) produces a high cathode resistance, limiting the electrochemical utilization and retention of the active material during cell cycling [7,9,13,14]. This high cathode resistance also impacts the extrinsic technological improvements possible for high-energy-density sulfur cathodes, which require both high sulfur utilization and a high amount of sulfur in the cathode (i.e., high sulfur loading and content) [14,15,16,17,18]. Hindered by the insulating nature of sulfur, previously reported sulfur cathodes often have low sulfur loading of <2 mg cm^−2^ and insufficient sulfur content of <60 wt% [19,20,21]. Regarding the active liquid-state materials, their electrochemical instability generally results from the formation of soluble lithium polysulfide species during the intermediate states of cell charging and discharging [10,11,12]. The polysulfides characterized by the formula Li_2_S_x_ with x = 4–8 are active liquid-state materials with strong chemical reaction activity and high solubility in the ether-based electrolyte currently used in lithium–sulfur batteries. The dissolved polysulfides tend to irreversibly diffuse out from the cathode and uncontrollably pollute the electrolyte and the active lithium-metal anode, which results in the loss of active material and poorer electrochemical reversibility [12,13,14]. Thus, the cell will eventually face a short cycle life, while an additional technological challenge is posed by the large amount of electrolyte in the cells. A high electrolyte-to-sulfur ratio is frequently reported in lithium–sulfur battery research, which requires a large amount of electrolyte to support the lithium-ion transfer because electrolyte is continuously contaminated by diffusing polysulfides and successively absorbed by the porous functional additives [4,5,6,7,20]. Moreover, while a large amount of electrolyte offsets the high cell resistance and slow reaction kinetics, it unfortunately causes the low energy density of lithium–sulfur batteries [4,5,6,7].

To address the problem posed by diffusing polysulfides, a functionalized separator is adopted to block the dissolved polysulfides and promote the reversible utilization of sulfur active material. Among the materials, nonpolar carbon materials majorly suppress the shuttle effect of polysulfide by the contribution of their porous structure. The porous structures and high specific surface area of carbon materials provide proper accommodation of the sulfur active material, thereby resulting in high sulfur utilization and improved electrochemical performance [22,23]. However, these properties also lead to a fast and large amount of electrolyte consumption; as a result, the reported high performance is mainly under conditions of high electrolyte-to-sulfur ratios. Herein, we investigate the use of carbon-coated separators as functional cell components, adopting their original function of separating the positive and negative electrodes while the carbon coating also blocks the rapid migration of the active liquid-state materials during cell cycling [15,16,17,18,19,20,21]. In the cell configuration, we considered a series of conductive carbon black materials as the coating materials, characterized by their unique nanoporosity and increased specific surface area. We also examined the effect of the nanopores and their resulting surface area on the polysulfide-trapping capability of the conductive carbon coatings. Moreover, we demonstrated the electrochemical analysis and performance improvements by adopting a high-loading sulfur cathode (sulfur loading and content of 4.0 mg cm^−2^ and 70 wt%, respectively) in the lean-electrolyte lithium–sulfur cell, with a low electrolyte-to-sulfur ratio of only 10 μL mg^−1^ [5,6,24]. Our experimental and analytical results demonstrate that the application of acetylene black as the coating material, with its limited porosity and small nanopore size, allows the high-loading sulfur cathode to attain high charge-storage and reversible capacities of 1112 mA∙h g^−1^ and 710 mA∙h g^−1^ after 200 cycles, respectively, under the lean-electrolyte condition. The high amount and utilization of sulfur also allow the cell with the acetylene black coating to achieve high areal and gravimetric capacities of 4.45 mA∙h cm^−2^ and 778 mA∙h g^−1^, respectively. The excellent electrochemical stability and long cycle life primarily result from the acetylene black coating inhibiting polysulfide diffusion while allowing steady lithium-ion transfer. Moreover, the nanoporosity of acetylene black addresses the issue of fast electrolyte consumption through the use of porous carbon coating materials in the lean-electrolyte cell.

## 2. Results and Discussion

### 2.1. Material Characteristics of Various Carbon Substrates and Module-Designed Carbon-Coated Battery Separators

**Figure 1:**
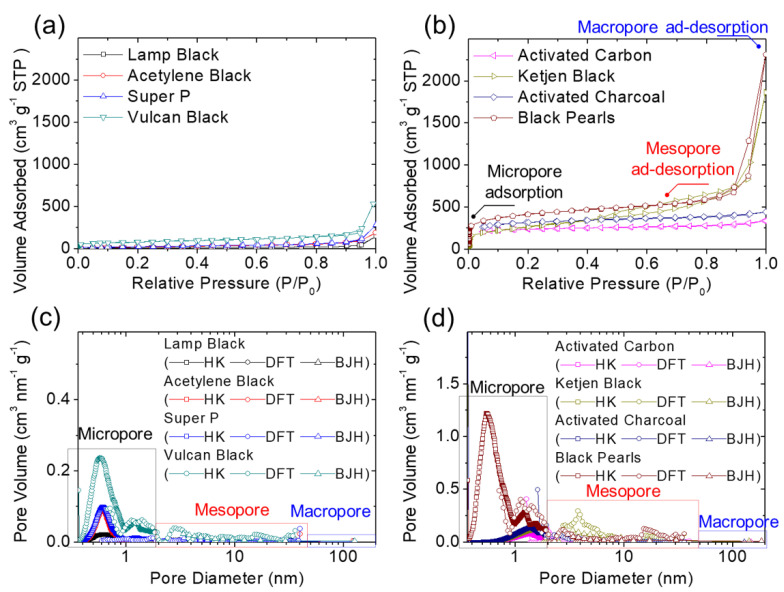
Material characteristics: (**a**,**b**) nitrogen adsorption/desorption isotherms and (**c**,**d**) pore-size distributions of various carbon materials for the carbon-coated separators.

**Figure 2:**
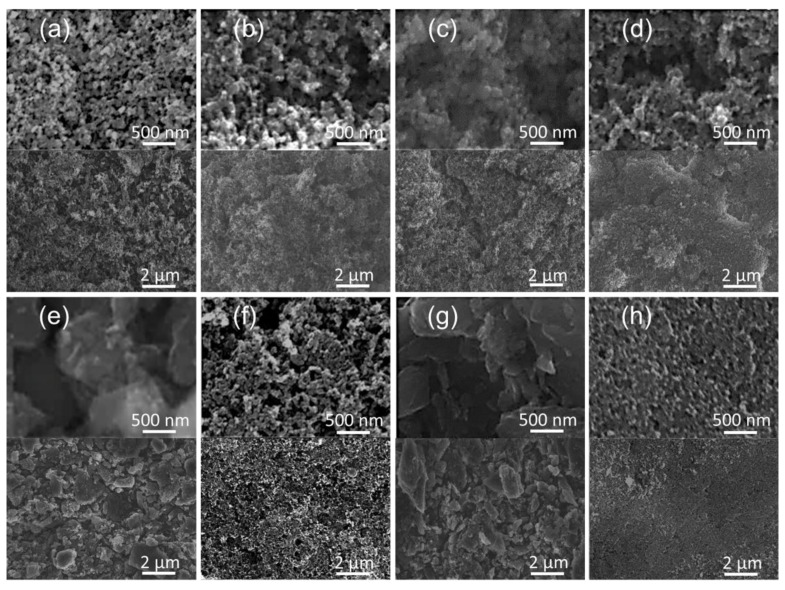
Material characteristics: scanning electron microscopy of (**a**) lamp black, (**b**) acetylene black, (**c**) Super P, (**d**) Vulcan black, (**e**) activated carbon, (**f**) Ketjen black, (**g**) activated charcoal, and (**h**) Black Pearls for the carbon-coated separators.

Figure 1 and Figure 2 summarize the material characteristics of the carbon blacks and their corresponding module-designed carbon-coated battery separators. Figure 1a,b show the nitrogen adsorption/desorption isotherms of all the carbon blacks studied. The analytical results of the isotherms indicated that lamp black, acetylene black, and Super P carbon materials were characterized by similar nonporous properties (Figure 1a), with Vulcan black also displaying strong adsorption behavior in its micropores and macropores. By contrast, a group of high-porosity carbon materials displayed strong micropore adsorption with an adsorption/desorption loop and a tail that are associated with their mesopores and macropores, respectively (Figure 1b). The activated carbon and activated charcoal displayed reversible type I isotherms, which demonstrates physisorption on microporous solids featuring relatively low external surface areas [25,26,27]. Ketjen black and Black Pearls demonstrated both the micropore adsorption behavior indicated by type I isotherms and the mesopore hysteresis loops characteristic of type IV isotherms [25,26,27], which proves that microporosity and mesoporosity were present. Figure 1c,d depicts the analyses of the porosity and pore-size distribution. The nonporous carbon blacks (i.e., lamp black, acetylene black, and Super P) displayed nonporosity and limited pore volume; however, the Vulcan black did feature a certain quantity of micropores (Figure 1c). The type I physisorption on the activated carbon and activated charcoal resulted from the adsorption and desorption capacity of micropores, with the pore size peaking at 1.4 nm (Figure 1d). Analysis of the porous carbon pore-size distributions [27,28,29,30,31,32,33] in Figure 1d also reveals that Ketjen black and Black Pearls demonstrated different nanopores and pore sizes in the pore-size distribution. Specifically, Ketjen black mainly possesses mesopores with pore size of 3.8 nm, while Black Pearls mainly comprises micropores featuring pore sizes of 0.5 nm, 1.1 nm, and 1.4 nm, with a minor mesopore peak at 15 nm resulting from stacks of Black Pearls nanoparticles.

Table 1 shows the BET specific surface area and total pore volume (in parentheses) calculated for the different conductive carbon materials, which gives values of 34.78 (0.23), 82.36 (0.28), 89.98 (0.43), 298.29 (1.02), 732.23 (0.52), 949.88 (2.91), 1002.82 (0.69), and 1320.95 m^2^ g^−1^ (3.61 cm^3^ g^−1^) for lamp black, acetylene black, Super P, Vulcan black, activated carbon, Ketjen black, activated charcoal, and Black Pearls, respectively. Of these conductive carbon materials frequently used in cathode fabrication [14,15,16,17,18,19,20,21,33], lamp black, acetylene black, and Super P had low specific surface areas of approximately 100 m^2^ g^−1^ and total pore volumes of less than 0.5 cm^3^ g^−1^, categorizing them as nonporous carbon materials. Moreover, no micropore adsorption behavior was detected in these three conductive carbon materials. Of these nonporous carbon materials, acetylene black showed a relatively high surface area, low pore volume, and small pore size. Vulcan black possessed a relatively high specific surface area and pore volume, with one third of the nanoporosity contributed by micropores. The other conductive carbon materials (i.e., activated carbon, Ketjen black, activated charcoal, and Black Pearls) exhibited large specific surface areas and high total pore volumes ranging from 732.23 m^2^ g^−1^ to 1320.95 m^2^ g^−1^ and from 0.52 cm^3^ g^−1^ to 3.61 cm^3^ g^−1^, respectively. Detailed analysis revealed that activated carbon and activated charcoal both had high microporosities of 80% and 75%, respectively, which contributed to their high surface area values. The high proportions of micropores in activated carbon and activated charcoal are also associated with their small average pore sizes of 2.89 nm and 2.79 nm, respectively. With a similarly high micropore adsorption performance, Black Pearls nanoparticles form clusters that produce its large average pore size of 10.96 nm, with 43% of its high surface area contributed by the detected mesopores and macropores. However, mesopores dominate the high porosity of Ketjen black. Therefore, we can categorize these conductive carbon materials as nonporous carbon materials (i.e., lamp black, acetylene black, and Super P) and porous carbon materials (i.e., Vulcan black, activated carbon, Ketjen black, activated charcoal, and Black Pearls), split into micropore-dominated (i.e., activated carbon and activated charcoal) and mesopore-dominated (i.e., Ketjen black) materials. The unique porosities of these conductive carbon materials enable us to explore the relative advantages of using them for the carbon coating of battery separators for high-performance high-loading sulfur cathode electrochemistry.

Figure 2 shows the observed morphology and microstructure of the module-designed carbon-coated separators for all of the different conductive carbon coatings. Each nanosized conductive carbon material was coated onto one side of a polymeric separator by the tape-casting method. These materials have clearly each formed functional layers with similar morphologies of accumulated conductive carbon nanoparticles. These carbon-coated battery separators were assembled into separate modules with sulfur cathodes, as described in Section 3.2, for electrochemical analysis.

### 2.2. Electrochemical Analysis of Various Carbon Substrates and Module-Designed Carbon-Coated Battery Separators

**Figure 3:**
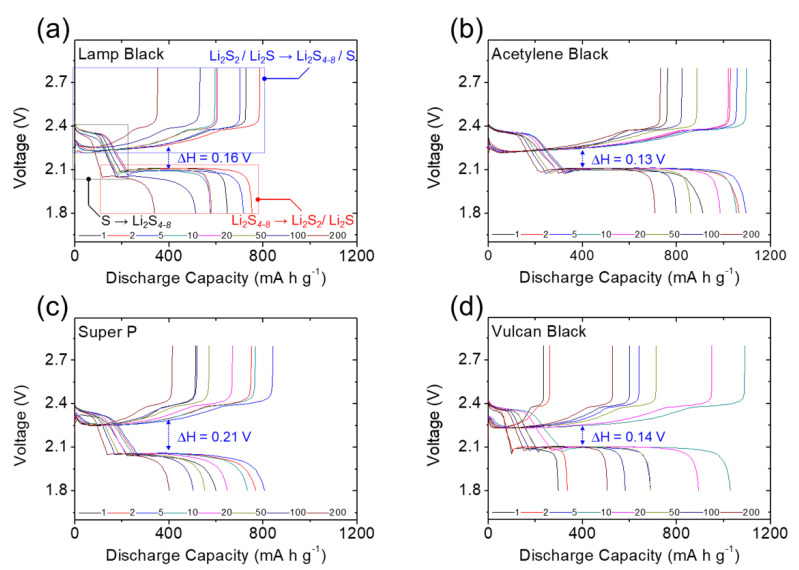
Electrochemical analysis: galvanostatic charge/discharge voltage profiles of the high-loading sulfur cathode in the lean-electrolyte cells with different carbon coating modules of (**a**) lamp black, (**b**) acetylene black, (**c**) Super P, and (**d**) Vulcan black.

**Figure 4:**
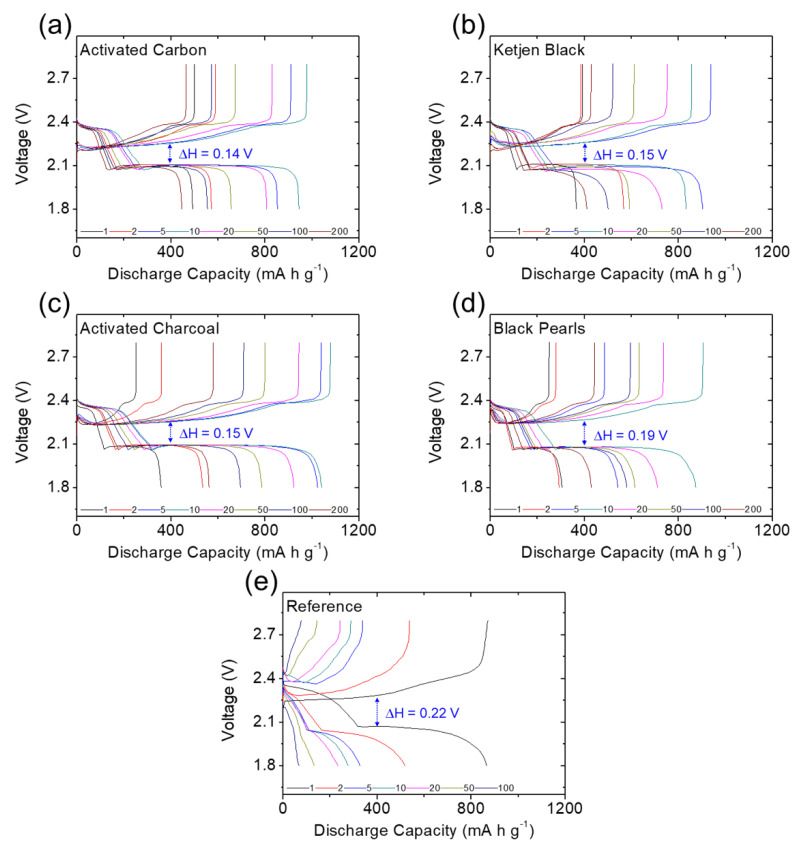
Electrochemical analysis: galvanostatic charge/discharge voltage profiles of the high-loading sulfur cathode in the lean-electrolyte cells with different carbon coating modules of (**a**) activated carbon, (**b**) Ketjen black, (**c**) activated charcoal, (**d**) Black Pearls, and (**e**) an uncoated reference cell.

Figure 3 and Figure 4 present the galvanostatic charge/discharge voltage curves of the lithium–sulfur cells equipped with the module-designed carbon-coated separator for long cyclability of 200 cycles at a cycling rate of C/10. All cells demonstrated two typical reduction reactions during discharging from 2.8 V to 1.8 V. The upper and lower discharge plateaus are attributed to the reduction of solid-state sulfur to liquid-state higher-order lithium polysulfides (Li_2_S_8_–Li_2_S_4_), followed by subsequent reduction to solid-state lower-order lithium polysulfides (Li_2_S_2_/Li_2_S) [13,14,15]. Thus, the first reduction involved the formation of liquid-state polysulfides with high solubility in liquid electrolytes and high mobility in cells [10,11,12,13], while the second reduction corresponds to the slow formation of insulating solid-state lithium sulfides [7,8,9,14]. These are the two main intrinsic material issues that should be addressed when designing advanced lithium–sulfur battery cathodes. In Figure 3 and Figure 4a–d, the complete upper and lower discharge plateaus of the cells with the carbon-coated separators illustrate the strong polysulfide retention and facile charge transfer with the different carbon coatings [8,9,10]. In contrast, the uncoated reference cell showed declines in both the upper and lower discharge plateaus during cycling (Figure 4e), resulting from the rapid loss of active liquid-state materials and poor utilization of insulating active solid-state materials [7,8,9,14]. This demonstrates the improved electrochemical stability and utilization provided by the carbon-coated separators.

In the oxidation reaction—reversibly charged from 1.8 V to 2.8 V—the corresponding charge plateaus are associated with the oxidation of lower-order and higher-order lithium polysulfides to solid-state sulfur [10,11,12,13]. The oxidation also relates to the polysulfide diffusion and inefficient utilization of sulfur. As shown in Figure 3 and Figure 4a–d, the carbon-coated separators enabled the high-loading sulfur cathode to maintain overlapping charge curves during continuous cycling and limited the irreversible capacity loss in each cycle, demonstrating the system’s excellent electrochemical stability and reversibility [11,12,13,14]. In comparison, the reference cell demonstrated the typical loss of active material and reaction capability with its higher charge plateaus (Figure 4e).

By considering the voltage hysteresis of the charge and discharge voltages, we determined the cell polarization attributable to the slow reaction kinetics and high electrode resistance caused by the high sulfur content in the cathode [13,14]. As shown in Figure 3 and Figure 4, the cells with the carbon-coated separators demonstrated stable and low polarization of 0.13–0.21 V during long-term cycling. The low polarization indicates that the carbon coatings would improve the charge transfer of high-loading sulfur cathodes, while maintaining steady lithium-ion transfer through the separator region (Figure 3 and Figure 4a–d). Among the carbon-coated separators, the module with the acetylene black coating had the lowest voltage hysteresis and the most stable charge/discharge curves, which demonstrate its outstanding electrochemical efficiency and stability. These results demonstrate the superior electrochemical performance of the high-loading sulfur cathode in the lean-electrolyte lithium–sulfur cell with the acetylene-black-coated separator. This implies that a nonporous carbon black coating with a low surface area and limited pore volume would be the most suitable coating material to boost cell performance, while avoiding the risk of absorbing too much liquid electrolyte from the coating layer (Figure 3b). By contrast, the reference cell faced increasing polarization during cycling, with the upper discharge plateau disappearing (Figure 4e). The increasing polarization may have been caused by redeposition of the diffusing polysulfides on the electrode, which would form an insulating solid-state material with no connection to the conductive network in the cathode [6,7,8,9].

**Figure 5:**
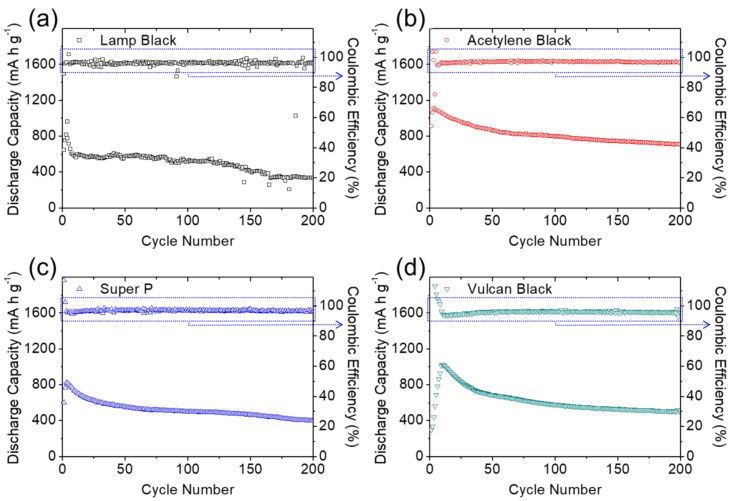
Electrochemical analysis: long-term cyclability of the high-loading sulfur cathode in the lean-electrolyte cells with different carbon coating modules of (**a**) lamp black, (**b**) acetylene black, (**c**) Super P, and (**d**) Vulcan black.

**Figure 6:**
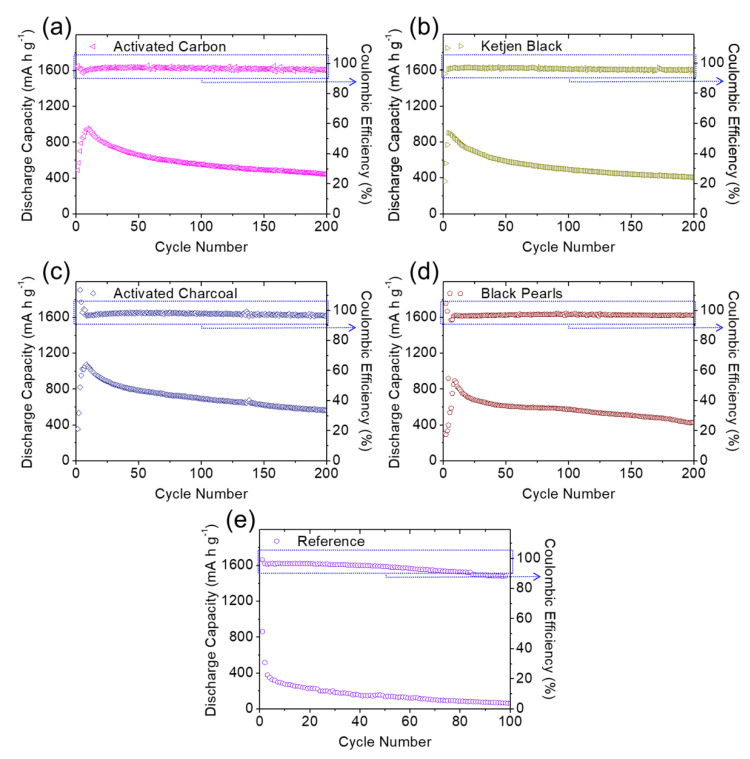
Electrochemical analysis: long-term cyclability of the high-loading sulfur cathode in the lean-electrolyte cells with different carbon coating modules of (**a**) activated carbon, (**b**) Ketjen black, (**c**) activated charcoal, (**d**) Black Pearls, and (**e**) an uncoated reference cell.

Figure 5 and Figure 6 display the electrochemical cyclability of the high-loading sulfur cathode in the lean-electrolyte lithium–sulfur cells with and without the carbon-coated separator for long cyclability of 200 cycles at a cycling rate of C/10. The module design of the coated separator with various conductive carbon materials boosted the overall cell performance, with enhanced electrochemical utilization and stability compared with the reference lithium–sulfur cells. The peak charge-storage capacity and reversible capacity values after 200 cycles (in parentheses) of the cells were 815 (338), 1112 (710), 826 (400), 1030 (505), 955 (445), 904 (410), 1077 (562), and 895 mA∙h g^−1^ (429 mA∙h g^−1^) for the modules coated with lamp black, acetylene black, Super P, Vulcan black, activated carbon, Ketjen black, activated charcoal, and Black Pearls, respectively. The high discharge capacity values correspond to the high electrochemical utilization of 50–70% of the large amount of insulating sulfur, which attained excellent areal and gravimetric capacities of 3.26–4.45 mA∙h cm^−2^ and 570–778 mA∙h g^−1^, respectively, depending on the cathode. After cycling, the cells also exhibited high capacity retention of 41–64%, confirming the enhanced electrochemical stability brought about by the carbon-coated separator. In comparison, the reference cell demonstrated a corresponding peak charge-storage capacity of 865 mA∙h g^−1^, but failed after 50 cycles while showing a decreasing Coulombic efficiency. The detailed electrochemical characteristics are summarized in Table 2.

Of all the modules, the carbon-coated separator with acetylene black simultaneously exhibited the highest peak charge-storage capacity (1112 mA∙h g^−1^), reversible capacity (710 mA∙h g^−1^), and charge/discharge efficiency, while also demonstrating the highest electrochemical utilization, reversibility, and stability. The acetylene-black-coated separator also allowed the high-loading sulfur cathode (with sulfur loading of 4.0 mg cm^−2^) to deliver the highest areal and gravimetric capacities of 4.45 mA∙h cm^−2^ and 778 mA∙h g^−1^, respectively; this performance is sufficient to power an electric vehicle [1,2,3,8,9,10,11]. Furthermore, desirable cycling performance resulted from the application of carbon-coated separators in the lithium–sulfur batteries, where the coating layers acted as polysulfide traps, considerably reducing polysulfide migration [10,11,12,13,14]. Coating layers with good conductivity would further function as the upper current collector, improving the charge transfer and reaction kinetics [6,7,8,9]. Moreover, the use of nonporous acetylene black, with its low specific surface area, low pore volume, small pore size, and absence of micropores, avoids the fast consumption of liquid electrolyte in the cell, maintaining the lean-electrolyte cell’s excellent cyclability and high Coulombic efficiency [8,11,20].

Although the acetylene-black-coated separator demonstrated the best cell performance, it is instructive to examine how the porosity of the conductive carbon materials influences the electrochemical performance of lithium–sulfur cells considering the high amount of active material in the lean-electrolyte cell. We considered the two categories of conductive carbon materials: nonporous carbon materials (i.e., lamp black, acetylene black, and Super P) and porous carbon materials (i.e., Vulcan black, activated carbon, Ketjen black, activated charcoal, and Black Pearls). The nonporous carbon coatings formed a closely connected carbon network on the polymeric separator that trapped the diffusing polysulfides, transferred ions and electrons to maintain a facile redox reaction, and avoided the fast consumption of liquid electrolyte, which improved the cycling stability and kept it in the lean-electrolyte condition. However, the lowest surface area and large pore size of the nonporous material lamp black resulted in limited improvements due to its relatively poor polysulfide-trapping capability, which was evidenced in its unstable discharge/charge efficiency. In contrast, the porous carbon blacks could be expected to enhance polysulfide trapping with their high surface area and large pore volume. However, the highly porous structure would also absorb and consume the liquid electrolyte, disrupt the steady and continuous lithium-ion transfer in the cell during conversion of active solid-state and liquid-state materials, and lead to a drop in discharge/charge efficiency. This would lead to inefficient activation processing and relatively fast capacity fading for porous-carbon-coated separators in lithium–sulfur cells.

## 3. Materials and Methods

### 3.1. Materials Characterization

Nitrogen adsorption/desorption isotherms and porosity analysis of the conductive carbon black materials were examined from 10^−5^ to 10^0^ P/P_0_ at 77 K by a high-resolution gas sorption system (autosorb iQ-MP/MP, Anton Paar, Austria). The nanoporosity (i.e., the specific surface area, total pore volume, and average pore size) was characterized by the Brunauer–Emmett–Teller (BET) and T-plot methods [25,26,27]. Pore-size distributions in the micropore (≤2 nm), mesopore (2–50 nm), and macropore (≥50 nm) ranges [25] were analyzed by the Horvath–Kawazoe (HK) method for the pore-size range of 0.3–2.0 nm [28,29], density functional theory (DFT, with an NLDFT equilibrium model based on slit pores) method for the pore-size range of 2.0–50 nm [27,30,31], and the Barrett–Joyner–Halenda (BJH) method for the pore-size range of 4.0–150 nm [27,32]. The morphology and microstructure of the carbon coatings were characterized by a field-emission scanning electron microscope (FE-SEM, SU-5000, HITACHI, Tokyo, Japan). Various conductive carbon blacks were used for the separator coatings, including lamp black (CABOT), acetylene black (Alfa Aesar), Super P (MTI), Vulcan black (Fuel Cell Store), activated carbon (CABOT), Ketjen black (Fuel Cell Store), activated charcoal (Alfa Aesar), and Black Pearls (CABOT). With these coatings, we studied the relationship between the nanoporosity of the module-designed carbon-coated battery separators and the electrochemistry of the resulting lithium–sulfur batteries.

### 3.2. Electrochemical Characterization

The carbon-coated separators were fabricated by the tape-casting method, using a doctor blade to coat a monolayer polypropylene membrane battery separator (2500, Celgard) with the conductive carbon coating layer. Specifically, conductive carbon blacks and polyvinylidene fluoride (PVDF) binder with a weight ratio of 9:1 were dispersed into *N*-methylpyrrolidinone (Sigma-Aldrich) to form a slurry. This slurry was coated onto one side of the separator with the doctor blade, then dried in a vacuum oven at 50 °C overnight. The as-prepared carbon-coated separator, with thicknesses of 13 ± 2 µm and areal loadings of 0.2 ± 0.01 mg cm^−^^2^, was then cut into a disk with a diameter of 1.9 cm which maintained the excellent flexibility and foldability of the polypropylene membrane. The carbon-coated separator was configured to face the sulfur cathode in the lithium–sulfur cell, which was assembled with the sulfur cathode, carbon-coated separator, and lithium anode (Sigma-Aldrich) placed in sequence. The sulfur cathode was made by mixing sulfur, conductive carbon black, and PVDF binder at a weight ratio of 70:15:15. The mixture was dispersed into *N*-methylpyrrolidinone to form a slurry, tape-cast on an aluminum foil, and dried at 50 °C overnight without a subsequent calendering process. The obtained sulfur cathode was cut into a disk with a diameter of 1.2 cm and a thickness of 100 µm that contained a high sulfur loading of 4.0 mg cm^−^^2^ and high sulfur content of 70 wt%. With consideration of the weight of the carbon coating, the sulfur content was 68 wt%. The electrolyte contained 1.0 M lithium bis (trifluoromethanesulfonyl)imide (Sigma Aldrich) and 0.1 M lithium nitrate (Alfa Aesar) in 1,3-dioxolane:1,2-dimethoxyethane (1:1, Alfa Aesar) solution. In the lean-electrolyte lithium–sulfur cell, the electrolyte-to-sulfur ratio was fixed at only 10 μL mg^−1^, and the reference cell was assembled in the same configuration with an uncoated polymeric separator. Galvanostatic charge/discharge tests were conducted at 1.8–2.8 V using battery-test instruments (Arbin Instruments, College Station, TX, USA) at a cycling rate of C/10 (1 C = 1675 mA g^−^^1^) to measure the charge/discharge voltage profiles, polarization, cyclability, areal capacity, and gravimetric capacity of the high-loading sulfur cathode with and without carbon coating of the separators. Without specific discussion, the discharge capacity was calculated based on the mass of sulfur. Areal capacity and gravimetric capacity in our research were based on the cathode dimensions with the consideration of sulfur loading and sulfur content, respectively.

## 4. Conclusions

We systematically studied the effect of the nanoporosity of carbon-coated separators on lithium–sulfur battery cathode electrochemistry and performance. The design of the carbon-coated separators used a range of nonporous and porous conductive carbon materials to optimize improvements to the electrochemical utilization and stability of the high-loading sulfur cathode. During long-term cycling of lean-electrolyte lithium–sulfur cells, the module design with an acetylene-black-coated separator, with its low surface area, low pore volume, and small pore size, achieved the best electrochemical utilization, stability, and efficiency. This cell design demonstrated promising electrochemical performance for long cycling of 200 cycles, with capacity retention of 64%, and high areal and gravimetric capacities of 4.45 mA∙h cm^−2^ and 778 mA∙h g^−1^, respectively—superior to the current state-of-the-art of lithium–sulfur cells reported in the literature.

## Figures and Tables

**Table 1:** Materials characteristics: porosity analysis of the carbons.

Carbon Sample	Surface Area (m^2^ g^−1^)	Pore Volume (cm^3^ g^−1^)	Microporosity		Pore Size (nm)
			Surface Area (m^2^ g^−1^)	Pore Volume (cm^3^ g^−1^)	
lamp black	34.78	0.23	0	0	26.11
acetylene black	82.36	0.28	0	0	14.13
Super P	89.98	0.43	0	0	19.61
Vulcan black	298.29	1.02	84.82	0.04	13.77
activated carbon	732.23	0.52	584.67	0.31	2.89
Ketjen black	949.88	2.91	57.85	0.03	12.29
activated charcoal	1002.82	0.69	754.07	0.39	2.79
Black Pearls	1320.95	3.61	752.47	0.40	10.96

**Table 2:** Electrochemical characteristics: cell performance of the high-loading sulfur cathode in the lean-electrolyte cells with different carbon coating modules.

Carbon Sample	Discharge Capacity (mA∙h g^−1^)	Reversible Capacity (mA∙h g^−1^)	Capacity Retention (%)	Cycle Number	Areal Capacity (mA∙h cm^−2^)	Gravimetric Capacity (mA∙h g^−1^)
lamp black	815	338	41	200	3.26	570
acetylene black	1112	710	64	200	4.45	778
Super P	826	400	48	200	3.31	579
Vulcan black	1030	505	49	200	4.12	721
activated carbon	955	445	47	200	3.82	669
Ketjen black	904	410	45	200	3.61	632
activated charcoal	1077	562	52	200	4.31	754
Black Pearls	895	429	48	200	3.58	627
reference	865	65	<10	100	3.46	605
Areal capacity and gravimetric capacity were based on the cathode dimensions with consideration of sulfur loading and sulfur content, respectively.						

## Sample Availability

Samples of the compounds are available from the authors.
